# Explaining the successes and failures of tuberculosis treatment programs; a tale of two regions in rural eastern Uganda

**DOI:** 10.1186/s12913-019-4834-2

**Published:** 2019-12-19

**Authors:** Jonathan Izudi, Imelda. K. Tamwesigire, Francis Bajunirwe

**Affiliations:** Department of Community Health, Faculty of Medicine, Mbarara University of Science and Technology, P.O. Box 1410, Mbarara, Uganda

**Keywords:** Barriers, Facilitators, Health systems strengthening, Treatment success, Tuberculosis, Uganda

## Abstract

**Background:**

Optimally performing tuberculosis (TB) programs are characterized by treatment success rate (TSR) of at least 90%. In rural eastern Uganda, and elsewhere in sub Saharan Africa, TSR varies considerably across district TB programs and the reasons for the differences are unclear. This study explored factors associated with the low and high TSR across four districts in rural eastern Uganda.

**Methods:**

We interviewed District TB and Leprosy Supervisors, Laboratory focal persons, and health facility TB focal persons from four districts in eastern Uganda as key informants. Interviews were audio recorded, transcribed verbatim, and imported into ATLAs.ti where thematic content analysis was performed and results were summarized into themes.

**Results:**

The emerging themes were categorized as either facilitators of or barriers to treatment success. The emerging facilitators prevailing in the districts with high rates of treatment success were using data to make decisions and design interventions, continuous quality improvement, capacity building, and prioritization of better management of people with TB. The barriers common in districts with low rates of treatment success included lack of motivated and dedicated TB focal persons, scarce or no funding for implementing TB activities**,** and a poor implementation of community-based directly observed therapy short course.

**Conclusion:**

This study shows that several factors are associated with the differing rates of treatment success in rural eastern Uganda. These factors should be the focus for TB control programs in Uganda and similar settings in order to improve rates of treatment success.

## Background

The overall goal of a tuberculosis (TB) control program is to ensure people with TB who are enrolled in the program complete treatment and become cured, endpoints collectively referred to as treatment success. Directly observed therapy short course (DOTS) is a global strategy for effective control of TB and its wider application has resulted into better treatment outcomes in several countries [1]. For instance, countries which have implemented all the five components of DOTS namely sustained political or government commitment, including political will at all levels, and establishment of a centralized and prioritized system of TB monitoring, recording and training; access to quality-assured sputum microscopy; standardized short-course chemotherapy for all cases of TB under proper case management conditions, including direct observation of treatment by a healthcare worker or community health worker for at least the first 2 months; uninterrupted or sustained supply of quality-assured drugs; and standardized recording and reporting system that allows assessment of treatment have reported high rates of cure and treatment completion [2]. In particular, the observation of people with TB as they take medication in real time by a treatment supporter or a healthcare provider ensures good adherence to and completion of treatment [3].

Although DOTS has been adopted and implemented widely, the achievement of the WHO desired treatment success rate (TSR) of at least 90% remains a challenge for most TB programs globally. Recent statistics suggest that the global TSR for newly diagnosed bacteriologically confirmed pulmonary TB (BC-PTB) has declined from 86% in 2014 to 83% in 2017 [4]. This decline presents an unprecedented concern for TB programs because of exacerbations in TB morbidity and mortality [5]. In addition, there is significant variation in TSR across sub Saharan Africa, both between and within countries. The between country variation is seen from TSR of 83.4% in Cameroon [6], 90.1% in Ethiopia [7], and 75.7% in Nigeria [8].

Uganda is an example of a country with significant variation in TSR. Overall, the country has a cure rate of 48% and a TSR of 80% [9], which are far below the desired WHO targets of 85% for cure rate and at least 90% TSR. In rural eastern Uganda, two districts of Serere and Ngora have higher TSR surpassing the WHO desired target of over 90% while the other two districts of Soroti and Kumi have at most 70% TSR, falling below the target. To date, information on factors shaping the variation in TSR across these districts in the same region is limited. The purpose of this study was therefore to explore factors associated with high and low TSR across districts in rural eastern Uganda. Exploring these factors is important for designing strategies and interventions that may be used to tackle hindrances to achieving high TSR while the good practices identified may be scaled up to improve TSR.

## Methods

### Study design and participants

We conducted a qualitative study and interviewed key informants to explore the successes and failures of the TB control program in rural eastern Uganda. The study participants consisted of health facility TB focal persons (TBFPs), District TB and Leprosy Supervisors (DTLS), and District Laboratory focal persons (DLFPs) from both settings, districts with low and high TSR. These participants were purposively sampled as key informants because they had rich-knowledge and experience in TB programing and the functioning of the district healthcare system and hence were expected to provide reliable and credible information [10]. We interviewed these participants until emerging ideas or responses were not any different from the ones earlier reported, suggesting saturation of ideas hence sufficient qualitative data as recommended [11].

### Study setting

This study was conducted in four predominantly rural districts in eastern Uganda, namely Soroti, Kumi, Ngora, and Serere. Soroti and Kumi districts have recently reported low TSR of 70 and 66.7% [12], while Ngora and Serere districts have high TSR of 90.9 and 95.3% [12], respectively. The districts were purposively selected to provide a framework for comparison of a well versus poorly performing district TB control program. The variation in performance for these TB control programs provides a basis and natural setting for our study. Altogether, the population in the four districts, located about 300 km from Kampala Capital City, is close to one million people. We enrolled participants from TB units with the highest patient loads in the selected districts. The estimated number of new people with TB in the study districts were 191 in Serere, 340 in Soroti, 313 in Kumi, and 121 in Ngora for the period July 2017 to June 2018 [13]. In Soroti district, the units included Soroti Regional Referral Hospital, Princess Diana Memorial Health Center (HC) IV, and Tiriri HC IV. A health center IV is a county level health facility in the structure of Uganda’s health system. The study sites in Kumi district included Kumi Hospital, Atutur Hospital, and Kumi HC IV. Kumi Hospital is a private not for profit health facility. The study sites in Ngora district were Ngora Hospital and Ngora HC IV and in Serere district, Serere and Apapai HC IVs were selected.

Each health facility has a TB diagnostic and treatment unit which provides TB services according to the WHO TB treatment guidelines [3]. At each TB unit, there is a TB focal person who is either a nursing, clinical, or medical officer to coordinate and provide TB services. However, technical leadership for TB management in the entire district is provided by the District TB and Leprosy Supervisor (DTLS), and each district has one. With respect to TB diagnostic services, each district also has a District Laboratory focal person (DLFP) to provide technical oversight.

### Data collection and quality control

Data were collected through audio-recording of key informant interviews which were conducted in English language in June 2019 at the selected health facilities. JI (a male public health specialist with experience in mixed-methods research) introduced the topics for the interview to the key informants and conducted the interviews, but was assisted by BK (a female social and public health specialist with 10 years of experience in qualitative research) to allow sufficient probing. The interview questions were asked in a non-sequential manner as the participant’s responses determined the next question to be asked, provided all the required topics were responded to at the end of the interview. All the key informant interviews were conducted in a quiet room and each lasted on average between 30 to 45 min. The key informant interview guide (Additional file 1) was specifically developed for this interview but was reviewed and approved by relevant research ethics committees, and pre-tested in the neighboring district of Katakwi to assess its suitability. All participants’ responses were transcribed within 48 h of completing data collection.

### Measurements

We asked the key informants questions on the following: current rates of treatment success at health facility and district levels, reasons for current performance of TB control programs; challenges facing the provision of TB services among healthcare workers; existing leadership and budget support to TB control program in the district; trainings and technical support supervisions on TB including the frequency, availability and logistical support to TB services, motivation of healthcare workers in providing TB services, and existing rewards and recognition strategies for good performing health facilities and individuals. We also asked questions on measures used in tracking performance of TB control program at district and health facility levels; and, strategies for improving rates of treatment success at both district and health facility levels.

### Data processing and analysis

JI transcribed the audio-recordings verbatim (word for word) in an iterative process within 48 h of completing the interviews to assure data integrity. During the transcription process, JI reviewed the transcripts for accuracy by playing back the audio-recordings while reading through the transcripts. In case of a discrepancy, the transcripts were edited to match the audio-recordings. The transcripts were then imported into ATLAS.ti for thematic content analysis using an inductive approach. Two authors (JI and IKT) independently conducted the analysis in three phases: data immersion, coding, and coding sort. In the first phase, the reviewers read the transcripts several times to achieve familiarity and to identify emerging issues. In the second phase, the authors flagged relevant parts of the transcripts with appropriate descriptive words (codes). In the third phase, the reviewers met and harmonized the independent codes. The harmonized codes were then combined to form categories, and the categories were combined to form emergent themes. A third independent reviewer (FB) verified the codes and themes. Themes were grouped by well performing districts versus poor performing districts as facilitators of treatment success or barriers to treatment success, respectively. We presented the results of the analysis in a summary table and used quotations to support the themes. The reporting of the study results adhered to the Consolidated Criteria for Reporting Qualitative Studies (COREQ) guidelines [14].

### Human subjects’ issues

All the participants provided written informed consent and were informed of their freedom to withdraw at any time during the interview. Our proposal received ethics review and approval from Mbarara University of Science and Technology Research Ethics Committee (Reference number: 03/11–18) and the Uganda National Council for Science and Technology (Reference number: HS 2531).

In addition, we obtained letters of administrative support from the respective District Health Offices and the Office of the President, Republic of Uganda (Reference number: ADM 194/212/01).

## Results

We approached 18 participants (10 TB focal persons, four DTLS, and four DTFPs) but reached saturation after 11 interviews (three DTLS, two DLFPs, and six TBFPs). Of the participants interviewed, six (two DTLS, three TBFPs, one DLFP) were from districts with lower level of TSR while five (one DTLS, three TBFPs, one DTLFP) were from districts with higher level of TSR. The mean age of the participants interviewed was 33.6 years (standard deviation, 4.78) and ranged from 27 to 34 years. Majority of the participants were males, and had worked for more than 5 years. Table 1 presents a summary of the participants’ characteristics.

**Table 1:** Participant socio-demographic characteristics

Characteristics	Level	Frequency (%)
Type of respondent	District TB and Leprosy Supervisor	3 (27.3)
	Laboratory focal person	2 (18.2)
	TB focal person	6 (54.5)
Participant distribution	District with low level of TSR	6 (54.5)
	District with high level of TSR	5 (45.5)
Respondent cadre	Clinical officer	4 (36.4)
	Laboratory technician	2 (18.2)
	Nursing officer	5 (45.4)
Sex	Female	3 (27.3)
	Male	8 (72.7)
Age category (years)	≤30	2 (18.2)
	> 30	9 (81.8)
	Mean (SD)	33.6 ± 4.78
	Range	27–34
District of respondent	Kumi	2 (18.2)
	Ngora	3 (27.3)
	Serere	2 (18.2)
	Soroti	4 (36.4)
Work experience (years)	< 5	3 (27.3)
	≥5	8 (72.7)
	Mean (SD)	4.82 ± 1.40
	Range	2–7 years

### Themes

Themes that were reported by respondents in districts with high treatment success were grouped as facilitators of treatment success while those in districts with low treatment success were classified as barriers to treatment success presented in Table 2.

**Table 2:** Emerging themes

Themes	Sub-themes
Facilitators of treatment success rate	• Use of data to make decisions and design interventions
	• Continuous quality improvement
	• Capacity building
	• Prioritization of better management of people with TB
Barriers to treatment success rate	• Lack of motivated and dedicated TB focal persons
	• Scarce and at times no funding for TB activities
	• Poor implementation of community-based DOTS

### Facilitators of treatment success rate

The facilitators of treatment success included the following themes: 1) Use of data to make decisions and design interventions; 2) Continuous quality improvement; 3) Capacity building; and 4) Prioritization of better management of people with TB.

### Use of data to make decisions and design interventions

Participants indicated that all the district TB units have performance targets that are usually set at the beginning of the year. To ease performance tracking and reporting, the targets are sub-divided on quarterly, monthly, weekly, and daily basis. At the end of each reporting time frame, data are collected and analyzed, and the performance for each health facility was tracked. To ensure data driven decision making, heath facility and district based TB review meetings were held so that health facility specific performances are shared and actions developed for each identified gap. The participants agreed that the usage of data in monitoring and evaluating the performance of the TB control program has improved the rates of treatment success in the districts.

*"During general staff meetings and sometimes end of monthly meetings we review our performance" (KI, High TSR district).*

*"The key thing we are doing is target setting per health facility because we give health facilities targets which we want them to achieve. Although some achieve and others do not for some reasons but the targets helps us a lot. Because you must do something when you know that this is what I must achieve. I give them [TB focal persons] targets per quarter, per month, and per week so that we can work on that" (KI, High TSR district).*

*"If a health facility does not achieve the quarterly targets, the balance is carried forward to the next quarter. After the end of the year, we tell the health facilities that you have fallen short of these numbers, but you have to look for this numbers in the next financial year" (KI, High TSR district).*

*"For example Serere HC [Health Center] IV, what we do, we have the TB Focal Persons with whom I share the findings of the health facility TB performance" (KI, High TSR district).*

On the other hand, participants reported that non-use of data to guide decision is associated with low rates of treatment success.

*"We do analyze TB data at the end of the quarter; but there has not been any platform for sharing the performance with health facilities. Because we have got nothing to do with district reviews, where we invite the DHTs and the health facilities to come and then we share TB performance, and where they can be given a chance to explain why they have performed poorly. That is why we have performed just like that [meaning low TSR] because there are no reviews" (KI, Low TSR district).*

### Continuous quality improvement (CQI)

CQI involves applying appropriate methods to close gaps between current and expected level of quality or performance as defined by standards. The aim is to systematically improve service quality by addressing gaps between current practices and desired standards. The Ministry of Health, Uganda requires all health facilities to have CQI teams constituted from existing staff members from each department and a community representative headed by the In-charge of the health facility or any member deemed fit. The role of the team is to address gaps in quality of health services provided to patients. At some health facilities, QI teams have been formed and are functional. However, at other health facilities, the CQI teams are either non-existent, non-functional, or existent but non-functional. Participants mentioned that performance gaps in TB care are common, and these are identified through regular data reviews but are addressed through the initiation of CQI projects. Some of the notable CQI projects were those that aimed at improving completion of sputum smear monitoring among people with TB.

*"Our data people collect the TB registers and they tell us our gaps. We then take actions such as starting a project [meaning quality improvement projects]" (KI, High TSR district)*

*"Actually, the issue of sputum follow-ups has been a big challenge. So I actually started a project [meaning QI projects] on sputum smear follow-ups and that’s why it has come up a little bit" (KI, High TSR district)*

### Capacity building

Building the competence of healthcare providers is important in delivery of quality services to people with TB. Notable capacity building opportunities include onsite mentorships, technical support supervisions, coaching, and offsite trainings. In this study, participants reported that regular supervisions to TB focal persons have helped to improve their capacity in providing better care to people with TB hence the improved the rates of treatment success in the districts. According to the participants, supervisions were conducted on quarterly basis by either the DTLS or members of the District Health Team during which TB focal persons are coached in a one-to-one session on the diagnosis and treatment of TB, and how to monitor response to TB treatment as well as how to record and compile TB data. The purpose is to improve the knowledge and competence of TB focal persons in providing quality services to people with TB. The TB focal persons gain more skills through practical interaction with their senior visiting colleagues.

"*At the district level, the DHO [District Health Officer] gives me a vote for technical support supervision although it is little money about 150,000 (One hundred fifty thousand Ugandan Shillings) every quarter. That means 50,000 [shillings] every month.*

*That means I am able to fuel my motorcycle, go to those underperforming health facilities" (KI, High TSR district).*

*"They [District Health Team members] regularly come around for technical support supervision, especially the District TB and Leprosy Supervisor" (KI, High TSR district).*

In addition, participants stated that targeted technical support supervisions were conducted alongside the regular technical support supervisions at poorly performing TB units in the district. The aim was to improve performance at poorly performing health facilities so as to match that of already well performing health facilities. These kinds of approaches were viewed to have associated with high rate of treatment success.

*"We [District Health Team] do quarterly support supervision to health facilities. Besides, we identify and support [meaning targeted technical support supervision] those health facilities which are performing badly" (KI, High TSR district).*

### Prioritization of better management of people with TB

This theme highlights an attitudinal shift to patient-centered approach in caring for people with TB. In districts with high treatment success rate, participants stated that in the past years, people with TB were not given priority by healthcare providers, but this had now changed because they receive services before other patients. Second, healthcare providers have positive attitudes towards people with TB and TB care in general. These reasons were emphasized to be associated with high rates of treatment success in the districts.

*"TB is now like respected; we appreciate that someone has TB and we do not treat them like before where TB was taken like a disease for people who are not hygienic, and where they kept blaming them. When they come for drugs they are given a priority, when they are coughing they are supported" (KI, High TSR district).*

*"Our health facility is not like other places [health facilities] where TB is like a neglected disease; we try to concentrate" (KI, High TSR district).*

Participants indicated that maximizing treatment completion and sputum smear microscopy monitoring while minimizing loss of people with TB across TB units in the districts are the main priority areas for improved rates of treatment success.

*"We are concentrating on treatment completion for now. Most of our TB patients actually complete treatment but the problem is sputum follow-ups. Our cure rate is still low although treatment success rate is high" (KI, High TSR district).*

*"All TB patients are monitored by sputum smears and encouraged to take their medications. Those who do not, we follow them up and make phone calls" (KI, High TSR district).*

Another priority mentioned relates to use of Village Health Team (VHTs), which was a common practice in districts with high rates of treatment success.

*"Another thing which is helping us is the use of VHTs [Village Health Team members] especially when TASO [The AIDS Support Organization] supports us we do. We use the VHTs to support us in looking for the lost TB patients so that the patient is brought to care to complete treatment" (KI, High TSR district).*

*"We use VHTs [Village Health Team Members] as our Ambassadors. In most cases, we ask the client to tell us which VHT is near him/her then the person will give use the names. On the other hand, when we meet with VHTs especially when they come to the ART [Anti-Retroviral Therapy] clinic, we attach them to the client nearer them so that they remind them to come for refills and also follow-up" (KI, High TSR district).*

However, in districts with low rates of treatment success, participants stated that the use of VHTs was less-commonly practiced.

*“We used VHTs at times for tracking TB patients and other things, but of course, you just have to rely on someone’s commitment or willingness which is a personal thing. You do not have control over them” (KI, Low TSR district).*

### Barriers to treatment success rate

We identified three themes as barriers to treatment success: 1) Lack of motivated and dedicated TB focal persons; 2) scarce and at times no funding for TB activities; and, 3) poor implementation of Community-based DOTS (CB-DOTS). In the subsequent paragraph, we discussed these themes into details.

### Lack of motivated and dedicated TB focal persons

The provision of TB services at health facility level to people with TB is by a TB focal person, an individual appointed by the head of the health facility. Such appointments are neither salaried nor receive special allowances but are added responsibilities. Participants reported that TB focal persons lack motivation and dedication in providing care to people with TB. This was viewed to have associated with low rates of treatment success in the districts. They firmly stressed that TB focal persons are inactive partly because most have not received orientation on their assigned roles and responsibilities.

*"TB focal persons are not active. Actually, they are not! Most of the TB focal persons right from the time they were told you are the TB focal person, they have never had any orientation. It is just an on job thing where they tell you to monitor this and that … .. But the trainings are not there" (KI, Low TSR district).*

One reason mentioned by participants for lack of motivated and dedicated TB focal persons was the absence of monetary rewards for good performance. Participants judged existing approaches for rewarding TB focal persons as not being useful at all.

*"When you perform well they only give you mere handclaps at the meeting. How useful is this?" (KI, Low TSR district).*

### Scarce and at times no funding for implementing TB activities

The lack of funds for implementing TB activities was a general problem in the region. This was reported by most participants to be associated with low rate of treatment success in the districts. It was mentioned that in certain occasions, limited funds for TB-HIV collaborative activities are provided, mostly by implementing partners. However, the funds are restricted to implementing HIV activities only. In addition, the funds are often submitted late to respective health facilities and this has resulted into TB focal persons using their own money in paying for transport charges incurred in the follow-up of people with TB.

*"At the district level, I basically rely on Implementing Partners only but from the DHT [District Health Team], there is nothing [meaning no funding] allocated to TB” (KI, Low TSR district).*

*"They [implementing partner] actually told us to first use the few resources we have thus to use personal money for transport to the patient's homes for follow up. After, you have to submit the report and then your money is refunded. But as I speak, we are not funded for doing TB outreach and follow ups. (KI, Low TSR district).*

*"The money you put in is refunded by TASO [The AIDS Support Organization], but only after you have submitted a report on the visits conducted. It is challenging because sometimes you don’t have the money readily available on you" (KI, Low TSR district).*

### Poor implementation of CB-DOTS

CB-DOTS, one of the five components of the WHO DOTS strategy for improving treatment adherence among people with TB was reported to be weak, non-functional, paper-based, and impractical. It was noted by most of the participants that the faults in the implementation of CB-DOTS is associated with low rate of treatment success in the districts.

*"You know the other bit is the functionality of CB-DOTS [Community-Based Directly Observed Therapy Short Course] is not there. Many times you will find it is on paper but not actual, according to the way the thing [DOTS] is formulated. It [DOTS] required that the healthcare worker visits the home of the TB patient, does the sensitization, and then selects someone to oversee the swallowing" (KI, Low TSR district).*

## Discussion

This study explored factors associated with low and high rates of treatment success across four districts in rural eastern Uganda. Our study identified the emergent themes as facilitators of and barriers to treatment success rate. The facilitators identified included use of data to make decisions and design interventions, continuous quality improvement, capacity building, and prioritization of better management of people with TB. Lack of motivated and dedicated TB focal persons, scarce and at times no funding for TB activities**,** and poorly implemented community-based DOTS were identified as barriers to treatment success.

This study identified that the use of TB data for decision making is associated with high rates of treatment success in the districts, suggesting local data is important in enhancing the performance of the health systems [15]. This corresponds with the requirements for a good and functional health information system which should produce, analyze, disseminate, and use reliable and timely information on health determinants, health systems performance, and health status [16]. It also conforms to the requirements of a health system in generating data, synthesizing information and promoting its availability and application [16]. This finding is consistent with results in a Zimbabwean study where the analysis and use of TB data by health facility and district staff led to improved quality of care to people with TB [17]. In particular, there was an increase in identification of persons with signs or symptoms suggestive of TB, new smear-positive TB cases, and reduced number of pulmonary TB cases without diagnostic smear results [17]. In contrast, when data are not used to monitor, evaluate, and improve performance of TB programs as was commonly noted in the poorly performing districts, low rates of treatment success emerges. The present finding hence suggest data are needed in achieving good health outcomes [18].

Participants indicated that capacity building through targeted and regular technical support supervisions is associated with high treatment success rate in the districts. According to the WHO, a well-performing health workforce should be available, competent, responsive, and productive in achieving the best possible health outcomes [16]. Capacity building through technical and targeted support supervisions hence improves the competence of healthcare providers and consequently leads to good health outcomes. This finding conform to the results of a preceding qualitative study where lack of in-service trainings for healthcare providers undermined the provision of quality TB services [19]. Elsewhere, lack of adequate knowledge among healthcare providers in managing TB and TB/HIV co-infected patients is reported to have reduced delivery of quality TB services [20] and poor treatment outcomes increased with knowledge inadequacy [21]. Our findings suggest that improving tuberculosis treatment outcomes may require the district and national TB control programs to deploy sufficient and competent health workforce.

Participants reported that continuous quality improvement (CQI) projects initiated to increase the completion of sputum smear monitoring is associated with high rates of treatment success. First, CQI is an exciting and promising intervention for closing performance gaps in healthcare [18]. Second, quality of healthcare mediates the relationship between the six WHO building blocks of health systems strengthening (service delivery, health work force, health information, health financing, leadership and, medical products, vaccines and technologies) and health outcomes (effectiveness, efficiency, responsiveness, social and financial risk protection) [22]. Accordingly, Uganda introduced CQI framework in 2005 to ensure health services provided to the population guarantee good quality of life and well-being at all levels of healthcare delivery [23, 24]. One of the principles of CQI is the objective use of data to identify performance gaps, analyze root causes of identified gaps, develop and implement improvement changes (solutions), and to monitor and evaluate health outcomes [25, 26].

As CQI is a deliberate effort in addressing low performance, it is not surprising that rates of treatment success improved. Although publications on use of CQI in TB control programs are scarce despite existence of TB specific CQI framework [18], in HIV programs, the use of CQI has remarkably improved early infant HIV diagnosis at 6 weeks [27], nutritional assessment among pregnant mothers [28], and retention of HIV positive adolescents in care [29]. Overall, it is important to recognize that certain districts have CQI teams while others do not. The explanation for this is anecdotal. First, the presence of CQI depends on the initiative of the local leadership. Second, CQI committees require additional resources which may not be available at some districts. Accordingly, the presence or absence of CQI teams at the different study sites might be due to these reasons.

Our data show that in districts with high rates of treatment success, Village Health Team members (VHTs) were commonly used to quickly trace and link people with TB who are lost to follow-up back into care. In these districts, VHTs volunteered to work despite absence of financial incentives because TB focal persons continually engaged them in activities of the health facilities. The aim of VHTs, a form of community health worker strategy introduced in 2002, is to link the community to the formal healthcare system [30]. VHTs are persons perceived as accessible, appropriate, and acceptable by members of the community since they are selected from amongst themselves [31], an important factor for good health outcomes. Therefore, our results support the involvement of VHTs in following up people on long term treatments [31]. In contrast, VHTs were not used in districts with low rates of treatment success due to financial constraints and their unwillingness to work without payments. Subsequently, these VHTs were not engaged by TB focal persons in TB related activities at the health facility. Controversy with VHTs is not unique to this study only.

In a previous study, VHTs received trainings and support from non-governmental organizations but failed to transition into the formal public healthcare system because they were entangled in power relations with the community and healthcare providers [32]. These kinds of transition failures degraded their roles. The same study observed that VHTs could not solve common problems such as lack of money for treatment, poor transport networks, and negative healthcare provider attitudes among others [32], factors that hinder people with TB from continuing with care. Therefore, although VHTS proved useful in TB care, strong coordination, leadership, and motivation are needed to get the most out of them. In our study, the lack of motivated and dedicated TB focal persons is associated with low rates of treatment success. This is in agreement with a former study where low staff motivation undermined the provision of good quality TB services in Uganda [19]. Similarly, a randomized control trial showed that people with TB who were allocated to a group where TB personnel were motivated have high treatment completion and sputum smear conversion rates compared to the group where TB personnel were not motivated [33].

This study reveals scarce or no funding for implementing TB activities is associated with low rates of treatment success. According to the WHO, a good health system should raise adequate funds for health in ways that ensure people use needed services, are protected from catastrophic expenses associated with having to pay for services, and provide incentives for healthcare providers and users [16]. Sufficient funding to TB programs is hence important for several reasons: training, monitoring and evaluation, supervision, staffing, follow-up of people with TB who are lost, and rewarding best performing individuals and health facilities among others. Lack of funding therefore undermines the health system’s capacity in providing quality TB services [20]. The absence of funds signifies weaknesses in budgeting for TB activities at district and national TB control program levels. Previous research highlighted weaknesses in planning as a significant operational barrier to delivery of quality TB services [34].

Another study reported that in resource limited settings, money is an important motivator since people expect payments for work done [35].

It emerged from participants that poor implementation of community-based DOTS, one of the five components of the WHO DOTS strategy, is associated with low rates of treatment success. Essentially, the successful implementation of DOTS requires a decentralized diagnostic and treatment network based on existing health facilities and integrated with primary healthcare, good program management based on accountability and supervision of healthcare workers, and an evaluation system for case-finding and cohort analysis of treatment outcomes [1]. The absence of any one of these principles might result into DOTS failure. Our interviews revealed that CB-DOTS had no established monitoring and evaluation system and lacked dedicated healthcare workers. These gaps were viewed to be associated with low rates of treatment success.

### Study strengths and limitations

Our study has several strengths. It is one of the few qualitative studies to explore factors associated with variation in rates of treatment success in a resource limited setting. Second, we explored the facilitators of or barriers to rates of treatment success from the perspectives of healthcare providers’ who hold rich-knowledge on the functioning of the healthcare system and delivery of health services in their respective districts. Third, we held the interviews until saturation was achieved which is important in exhaustively underscoring the reasons for the differing rates of treatment success across the districts.

Nonetheless, there are some limitations. This study is not exempt from social desirability or self-reporting biases although we attempted to minimize this by use of several probing questions.

This study was conducted in a rural setting so the findings may not be generalizable to urban settings. Another limitation is that we did not interview or obtain information from the District TB and Leprosy Supervisor and District Laboratory focal person for all participating districts. In addition, we did not interview a TB focal person at every health facility/site chosen in the four districts.

### Conclusions and recommendations

Our study shows that several factors are associated with variation in rates of treatment success across districts in rural eastern Uganda. The high TSR is associated with use of data to make decisions and design interventions, continuous quality improvement, capacity building, and prioritizing better management of people with TB. Reported barriers to TSR included lack of motivated and dedicated TB focal persons, scarce and at times no funding for TB activities**,** and a poorly functioning CB-DOTS program. These factors should be the focus for interventions to improve the district and national TB programs in rural Uganda and other settings.

## Supplementary information

**Additional file 1.** Key Informant Interview guide.

## Data Availability

The datasets used and/or analyzed during the current study are available from the corresponding author on reasonable request.

## Abbreviations

DLFP: District laboratory Focal Person
DTLS: District TB and Leprosy Supervisor
TB: Tuberculosis
TBFP: Tuberculosis Focal Person
TSR: Treatment Success Rate
