# Supplementary material for: Explaining the successes and failures of tuberculosis treatment programs; a tale of two regions in rural eastern Uganda
Source: BMC Health Serv Res. 2019 Dec 19;19:979. doi: 10.1186/s12913-019-4834-2 (PMC6923886; doi:10.1186/s12913-019-4834-2)
Supplement: Supplementary file 1 — Additional file 1. Key Informant Interview guide. [file 12913_2019_4834_MOESM1_ESM.docx]

**Key Informant Interview guide**

| **Instruction:**   1. This interview is designed to explore factors shaping TB treatment success rate in your district or health facility. 2. The interview will explore health facility and district level factors that shape treatment success rate. 3. The target participants are District Laboratory Focal Persons, and District TB and Leprosy Supervisors |
| --- |

Q1: Please describe some of the challenges that the district is faced with in tuberculosis (TB) care

Probe on;

- Logistical problems like sample transportations for laboratory tests
- Supplies and reagents: stock outs of anti-TB drugs, testing reagents, among others
- Human resources challenges: number of health workers and willingness to work in TB care
- Availability of registers for recording
- Patient level concerns: defaulting/getting lost, stigma and discrimination, completion of treatment

Q2: Share with me how the district is performing in TB care, and some of the reasons for the performance

Probe on;

- District TB performance indicator
- An assessment of the performance, whether satisfactory or not/ meeting standards or not
- Why poor or good performance

Q3: Tell me of any district or health facility specific leadership and commitment to TB care

Probe on;

- Presence or absence of district/health facility led TB performance reviews
- Action plans for TB care
- Budgetary support for TB care
- Coordination of TB activities
- Rewards/recognitions for best performing facilities or individuals
- Punitive measures such as transfers, demotions, among others for poor performance

Q3: Share with me the organization of the district/or health facility leadership for TB care

Probe on;

- The involvement of District Health Teams/ health facility staffs
- Steps taken to improve/maintain TB performance.

**Thank you for the participation**
